# Hydrophobic and Anti-Fouling Performance of Surface on Parabolic Morphology

**DOI:** 10.3390/ijerph17020644

**Published:** 2020-01-19

**Authors:** Yu Li, Shengke Yang, Yangyang Chen, Dan Zhang

**Affiliations:** 1 Key Laboratory of Subsurface Hydrology and Ecological Effects in Arid Region, Ministry of Education, Chang’an University, Xi’an 710054, China; ly18821763220@126.com (Y.L.); cgezcyy@126.com (Y.C.); 2017129063@chd.cn (D.Z.); 2 School of Water and Environment, Chang’an University, Xi’an 710054, China

**Keywords:** parabolic, hydrophobicity, anti-fouling, contact angle, oil–water separation

## Abstract

The hydrophobicity and anti-fouling properties of materials have important application value in industrial and agricultural production and people’s daily life. To study the relationship between the unit width L_0_ of the parabolic hydrophobic material and the hydrophobicity and anti-fouling properties, the rough surface structure of the parabolic with different widths was prepared by grinding with different SiC sandpapers, and further, to obtain hydrophobic materials through chemical oxidation and chemical etching, and modification with stearic acid (SA). The morphology, surface wetting and anti-fouling properties of the modified materials were characterized by SEM and contact angle measurement. The oil–water separation performance and self-cleaning performance of the materials were explored. The surface of the modified copper sheet forms a rough structure similar to a paraboloid. When ground with 1500 grit SiC sandpaper, it is more conducive to increase the hydrophobicity of the copper sheet surface and increase the contact angle of water droplets on the copper surface. Additionally, the self-cleaning and anti-fouling experiments showed that as L_0_ decreases, copper sheets were less able to stick to foreign things such as soil, and the better the self-cleaning and anti-fouling performance was. Based on the oil–water separation experiment of copper mesh, the lower L_0_ has a higher oil–water separation efficiency. The results showed that material with parabolic morphology has great self-cleaning, anti-fouling, and oil–water separation performance. The smaller the L_0_ was, the larger the contact angle and the better hydrophobic performance and self-cleaning performance were.

## 1. Introduction

Infiltration in life can be seen everywhere, for example, the excess energy consumed by a ship sailing due to hydraulic friction [1]. Interest in super-hydrophobic surfaces has increased in recent years [2,3,4,5,6]. Hydrophobicity and stain resistance are widely used in many areas such as anti-biofouling surfaces in a marine environment [7,8,9,10], fluid drag reduction [11,12], anti-contamination surfaces of windows for buildings and automobiles [13,14,15], microfluid [16,17], and many others. Therefore, studying hydrophobicity and stain resistance is particularly important in industry, agriculture, and people’s daily life.

To obtain excellent hydrophobic and anti-fouling materials, scholars constantly explore the prediction theory of the superhydrophobic model and the preparation technology. Nishino et al. [18] found that after a low surface energy modification on a smooth solid surface, the maximum contact angle (CA) did not exceed 120°. Therefore, the construction of the surface microtopography has a great influence on the hydrophobic surfaces. In terms of model theory prediction, by analyzing the microstructure of natural super-hydrophobic surface examples, based on Young’s theory [19], both the classical Wenzel model [20] and the Cassie–Baxter model [21] consider that the roughness of the solid surface can enhance the hydrophobicity of the surface. However, both the Wenzel model and the Cassie–Baxter model are only suitable for the case where the droplets are sufficiently large relative to the surface convex structure. In addition, Young’s equation, the Wenzel model and the Cassie–Baxter model provided a basis for the subsequent proposed hydrophobicity model. Shi et al. [22] established an energy model based on the minimum Gibbs free energy. Salvadori et al. [23] designed a periodic micropore array model. Eyal Bittoun and Abraham Marmur [24] constructed different morphologies of rough surfaces: a cylinder, a truncated cone, and a hemisphere.

After the investigation of the microstructure deeply, scholars found the key factors affecting the contact angle are the aspect ratio h/R of the microstructure, a/b ratio of the column width, and the multiscale microstructure. Nosonovsky [25] studied the relationship between the surface roughness and the wetting property of the hemispherically topped cylindrical structure, conical structure, and pyramidal structure, finally, it was found that as the aspect ratio h/r increased, both hemispherically topped cylindrical structure and pyramidal structure can reach the maximum contact angle. For the column width ratio a/b, Yamamoto and Ogata [26] thought that the microscopic rough surfaces were pillar surfaces and did not consider the case where the protrusive surfaces were curved. Zhang [27] constructed the top of the mastoid, which was a curved structure, and found that the microstructure parameters and the unit width of the mastoid were the key factors to affect the contact angle. These microstructures are used to improve the hydrophobicity of materials. However, previous studies have lacked an in-depth exploration of the prediction of hydrophobicity and stain resistance in the establishment of proprietary spherical or parabolic models.

In terms of material preparation, in recent years, inspired by surface structures like the lotus leaf self-cleaning surface and mosquito compound eyes [28], a series of superhydrophobic materials were prepared and used for oil–water separation. Some of the most used methods consist of the surface modification of metallic meshes and fabrics, with diverse techniques such as hydrothermal methods [29], leaching [30], electrochemical anodization [31], solution immersion [32,33], and thermal polymerization [34]. Li has done a series of studies on oil–water separation materials in harsh conditions [35], such as fabricating superhydrophobic CS and silica overlap coated meshes for efficient oil–water separation. Xue et al. [36] reported superhydrophobic polyester fabrics obtained through alkali etching, chemical vapor deposition, and ultraviolet light initiated click chemistry. Unfortunately, the component toxic fluorinated materials may cause great harm to the environment. Li et al. [37] suggest an innovative biomimetic way for the recycling of valuable inks or reactants, especially in environmental protection, chemical analyses, and printing processes. These materials have a great oil–water separation effect, however, the material’s anti-fouling performance was not considered.

Anti-fouling is a significant feature of hydrophobic materials. For example, Liu et al. [38] have developed a superhydrophobic and antifouling performance PET fabric, which still maintains great performance after being recycled. Li et al. [39] have demonstrated a novel electrostatic manipulation method to control the jumping of water drop in varied directions. This research would provide new opportunities to improve self-cleaning and reduce icing. Wang et al. proposed a dynamic, time-varying cleaning method based on superhydrophobic surfaces [40]. However, some shortcomings cannot be ignored, including long preparation process and expensive cost. Copper meshes have attracted widespread attention for their mechanical strength, low density, high specific surfaces, and environmentally friendliness [41,42]. Therefore, the technique used to etch a specific shape on the copper surface and then apply it as a hydrophobic and self-cleaning material was the most interesting issue.

In this paper, parabolic structure materials with different unit widths of the parabolic (L_0_) can be obtained by grinding, chemical etching, oxidation, and modifying. Then, the surface of the materials was characterized by scanning electron microscopy (SEM) and contact angle measurements. Finally, the copper mesh and copper sheets were applied to oil–water separation, anti-fouling, and self-cleaning experiments, which showed that the parabolic morphology has great hydrophobic and anti-fouling properties. It was shown that the parabolic morphology materials had certain application value in environmental protection.

## 2. Materials and Methods

### 2.1. Instruments and Chemicals

FEI Quanta 200 SEM (FEI company, Hillsboro, OR, USA), SL200KS contact angle meter (American Kono Industrial Co. Ltd., Seattle, WA, USA).

Copper meshes (200 meshes) were purchased from Shenyang Copper Network Co., Ltd. (Shenyang, China). Copper sheets were purchased from Tianjin Shengao Chemical Reagent Co., Ltd. (Tianjin, China). Acetone (purity > 99%), benzene (purity > 99%), ethanol (purity = 99.5%), stearic acid (SA), FeCl_3_ (35 wt %) (purity > 98%), H_2_O_2_ (30 wt %), and kaolin (purity > 99%) were purchased from Tianjin Kemiou Chemical Reagent Co., Ltd. (Tianjin, China). SiC sandpapers (320, 400, 600, 800, 1000, 1200, and 1500 grit) were purchased from Shanghai Ruihan Vision Co., Ltd. (Shanghai, China). The experimental water was deionized water. All other chemicals were analytical-grade reagents.

### 2.2. Preparation of Materials

Copper meshes (200 meshes) (4 cm × 4 cm) and copper sheets (2 cm × 4 cm) were placed in a beaker and ultrasonically cleaned in acetone, ethanol and deionized water for 10 min to remove oil and inorganic impurities on the surface. To achieve different unit width of copper meshes and copper sheets surface by mechanical abrasion, 320, 400, 600, 800, 1000, 1200, and 1500 grit SiC sandpaper were used to grind 20 times horizontally so that the scratches were aligned parallel to each other. The copper meshes and copper sheet samples were washed successively with absolute ethanol and deionized water and air-dried at room temperature. The cleaned copper meshes were placed in a well-prepared 35% FeCl_3_ solution and ultrasonically etched for 20 min with an ultrasonic cleaner; the etched copper meshes were placed in 30% H_2_O_2_ and oxidized by an ultrasonic cleaner for 3 min. The copper meshes were immersed in a 10 mol/L ethanolic solution of SA (1:3 refers to the volume ratio of ethanol to water) at 60 °C for 24 h. The cleaned copper sheets were immersed in a 10 mol/L ethanolic solution of SA (1:3 refers to the volume ratio of ethanol to water) at 60 °C for 30 min. The modified copper meshes and copper sheets samples were washed successively with absolute ethanol and deionized water and air-dried at room temperature.

### 2.3. Contact Angle Measurements

The water contact angles at ambient temperatures using an SL200KS contact angle meter. The test steps are as follows: Firstly, click the moving image button in the upper right corner of the interface. Then, place the sample to be tested on the stage, 3 μL liquid is extracted from the microsyringe and fixed above the sample, then turn the knob on behind the base of the camera, adjust the distance from the camera to the stage to make the image clearest. Subsequently, the volume of the test water droplets approximately 3 µL squeezed out from the microsyringe, in this time, a clear small droplet dropping from the syringe. Finally, keep the droplet on the sample, click the freeze image button in the upper right corner of the interface to save the picture, press the angle of the measurement button, and calculate the contact angle. At least three parallel positions on the surface were measured to obtain average contact angle values.

### 2.4. Oil–Water Separation Test

An oil–water mixture of benzene–water was used for the separation experiments. For convenience, deionized water was stained with methylene blue. Oils were stained with Sudan I. Oils and deionized water was mixed at a volume ratio of 1:1, respectively. The oil–water separation performance of the copper meshes was determined by the gravity-driven oil–water separation test.

### 2.5. Anti-Fouling and Self-Cleaning Experiments

A red suspension of kaolin-Rhodamine B was formed by a mixture between 15 g kaolin and rhodamine B solution for 30 min. Each sample was weighed by high precision balance and recorded as initial value M_0_. The copper sheets were immersed in the red suspension of the uniformly mixed kaolin-Rhodamine B. After soaking for 3 s, the copper sheets were placed obliquely to ensure that the angle with the horizontal plane was 45°. Finally, the copper samples were successively dried in air at room temperature and samples with soil were weighed again as Mx. The difference ΔM was the mass of the contaminant to which the sample surface adhered.

## 3. Results and Discussion

### 3.1. Material Surface of Different Unit Width of the Parabolic

To better observe the microscopic morphology of the surface of the copper sheets, it was necessary to carry out gold pretreatment on the surface of the sample before the test to enhance the conductivity of the sample and then fix the sample on the sample stage to observe the test. The operation needs to be done in a vacuum environment. The microscopic morphologies of the surface of the copper sheets after grinding were observed by SEM.

**Figure 1:**
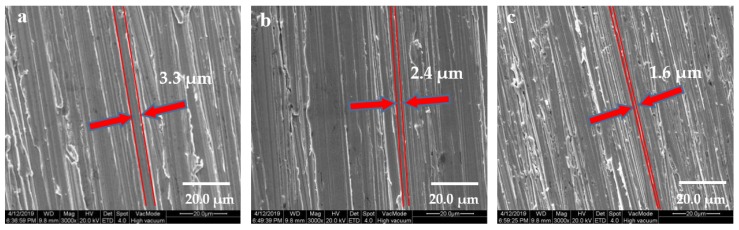
Copper sheets morphology under SEM. (**a**) Ground with 320 grit SiC sandpaper; (**b**) ground with 800 grit SiC sandpaper; (**c**) ground with 1500 grit SiC sandpaper.

Figure 1a–c showed that the copper sheets were ground with 320, 800, and 1500 grit SiC sandpapers, respectively. It can be seen via a microscope that the surface of copper sheets becomes rough. As can be seen from Figure 1, with the grit of the sandpaper increased, the unit width of the parabolic had a significant change. Figure 1a–c showed the different L_0_ were 3.3 μm, 2.4 μm, and 1.6 μm, respectively.

### 3.2. Hydrophobic Performance

The contact angle refers to the wetting property of water droplets on a solid surface. For hydrophilic surfaces, the droplets wet the surface easily and spread out. The contact angle of the water droplets is less than 90°. However, for hydrophobic surfaces, the droplets were not easily spread and appeared spherical on the surface, the contact angle of the water droplets is greater than 90° [43]. It is generally believed that as the contact angle increases, the hydrophobic property improves. Therefore, the contact angle is an important index to reveal hydrophobic and super-hydrophobic properties [44,45,46,47].

**Figure 2:**
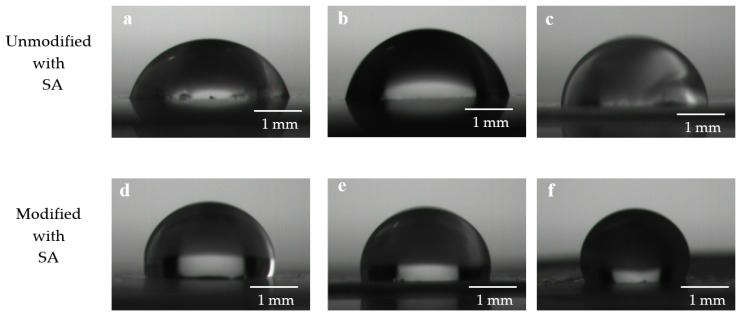
Wettability of water droplets on the surface of the copper sheets. (**a**,**d**) The contact angle (CA) of L_0_ = 3.3μm; (**b**,**e**) the CA of L_0_ = 2.4 μm; (**c**,**f**) the CA of L_0_ = 1.6 μm.

The wetting behaviors of water on the as-prepared copper sheets were evaluated by the contact angle measurement. The water contact angles of the copper sheets with the different unit width were shown in Figure 2. Figure 2a–c and Figure 2d–f were the CAs of copper sheets with unit width L_0_ 3.3 μm, 2.4 μm, and 1.6 μm, respectively. It can be seen from Figure 2a–c, the unmodified with SA copper sheets were hydrophilic and the CAs of the copper sheets surface were 68.7°, 72.5°, and 82.4°, respectively, which indicated that as the L_0_ of the copper sheets decreased, the CAs increased. The results of Figure 2 agree with the results from Shi et al. [22], who used different PMMA samples with convex width analytical methods.

In Figure 2d–f, the CAs of the as-prepared copper sheets that were modified with SA were measured to be 94.5°, 100.2°, and 110.5°, respectively, which showed that the copper sheets became a hydrophobic surface after modification with SA [48] and it was consistent with the research of Gui-Hua et al. [49]. It can be seen that as the L_0_ of copper sheets modified with SA decreased, the CAs increased.

To better illustrate the contact properties of water droplets on the copper sheets, all the contact angles were listed in Table 1.

From Table 1, the CA of the copper sheet modified with SA was always bigger than that of unmodified with SA. That was due to the low surface energy being a key factor in achieving superhydrophobicity [43]. The surface free energy can affect the value of the contact angle; the smaller the surface free energy is, the larger the contact angle is.

### 3.3. Oil–Water Separation Performance

Copper meshes can be obtained by grinding 320, 800, and 1500 grit SiC sandpaper, etching with 35% FeCl_3_, oxidation with 30% H_2_O_2_ solution, and modification with SA. Previous studies have shown that using SA as a modifier can form a super-hydrophobic membrane surface on the surface of the materials [50,51,52].

To understand the separation efficiency of the different unit width of the parabolic L_0_ of copper meshes surface for oil–water mixtures more intuitively, the oil–water separation experiment was carried out. The process is as follows: To completely cover the beaker mouth, the as-prepared copper meshes that were trimmed into dimensions of 4 cm × 4 cm were fixed over the beaker, and then the small beaker was placed in the center of the large petri dish. The volume of deionized water and benzene used for the experiments were both 6 mL. After shaking the mixture of oil and water in the syringe, then gradually squeezed the syringe and slowly dripped the mixture from the needle to the membranes.

**Figure 3:**
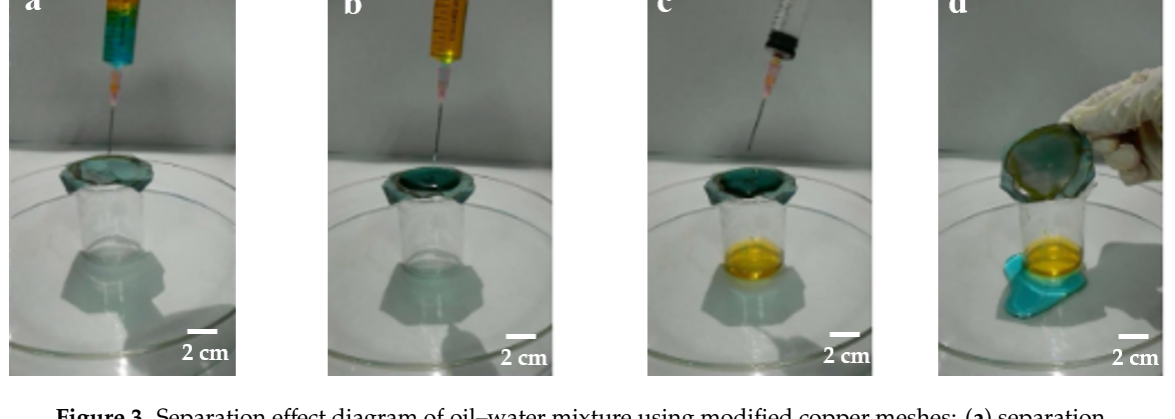
Separation effect diagram of oil–water mixture using modified copper meshes: (**a**) separation of preparation; (**b**) separation begins; (**c**) in separation; (**d**) end of separation.

A device diagram of the oil–water separation test is shown in Figure 3a. The as-prepared copper meshes completely covered the beaker mouth to prevent deionized water from entering the beaker from other places. The separation experimental processes of benzene–water are shown in Figure 3b. The density of benzene was less than water and water passed first through the syringe and then flowed into the membranes. With the accumulation of deionized water on the copper mesh surface, it can be found that water droplets gradually flowed out from the edge of the copper mesh and did not penetrate the copper mesh into the small beaker. However, benzene passed through the deionized water, reached the surface of the membranes, and flowed into the beaker quickly, as shown in Figure 3c. The process of oil–water separation in the whole process was approximately 3 min. Figure 3d showed that after completion of the experiment, there was almost no blue residue on the membranes by pouring the water above the membranes into a large petri dish.

**Figure 4:**
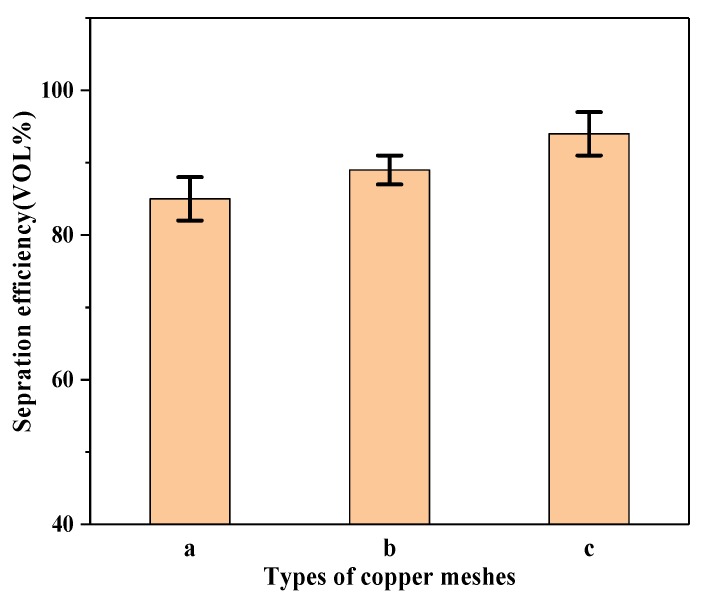
Results of the separation efficiency of copper meshes. (**a**) Ground with 320 grit SiC sandpaper; (**b**) ground with 800 grit SiC sandpaper; (**c**) ground with 1500 grit SiC sandpaper.

According to the above methods, the mixtures of benzene and deionized water were separated by the oil–water separation experiment. When the separation process was completed, the oils in the beaker were poured into a measuring cylinder and the volumes of oils were measured after separation. It was found that the volumes of benzene after separation were approximately 5.1 mL, 5.3 mL, and 5.6 mL, respectively. After separation of oil and water, according to the amount of oil passing through the membranes and the separation efficiency, *η* (%), was calculated by Equation (1).

(1) $$\eta =\frac{V_{t}}{V_{0}}\times 100\%,$$

where *V*_0_ is the benzene mass of the original and *V_t_* is the benzene mass after separation. The results obtained are shown in Figure 4.

The oil–water separation efficiency of the three different L_0_ copper mesh surfaces were measured. From Figure 4, the separation efficiency of benzene was 85%, 89%, and 94%. It can be seen that due to the different etching methods of copper meshes, the separation efficiency was different [53,54]. It was shown that with a decrease in L_0_, the separation efficiency became higher.

### 3.4. Anti-Fouling and Self-Cleaning Performance

**Figure 5:**
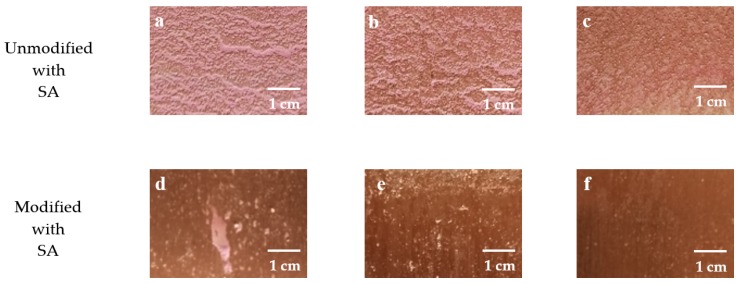
The self-cleaning effect diagram of the copper sheets surface. (**a**,**d**) Copper sheet of L_0_ = 3.3 μm; (**b**,**e**) copper sheet of L_0_ = 2.4 μm; (**c**,**f**) copper sheet of L_0_ = 1.6 μm.

**Figure 6:**
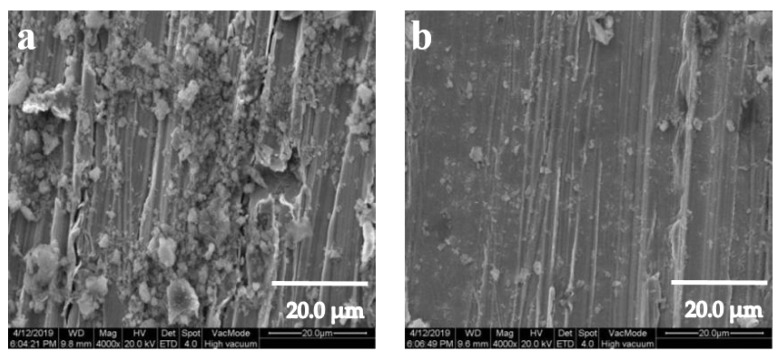
SEM image of self-cleaning on copper sheet surfaces: (**a**) unmodified with stearic acid (SA); (**b**) modified with SA.

To understand the self-cleaning properties of the untreated and as-prepared copper sheets more intuitively. The copper sheets were immersed in the red suspension of the uniformly mixed kaolin-Rhodamine B. The kaolin adhesion to the surface of copper sheets were observed by SEM. Figure 5 and Figure 6 showed the self-cleaning performance of soil on copper sheets with or without modification with SA. Figure 5a–c and Figure 5d–f were the copper sheets that were ground with 320, 800, and 1500 grit SiC sandpapers, respectively. As the L_0_ of the copper sheet surfaces decreased, the amount of kaolin gradually decreased, which indicates that copper sheets had better self-cleaning properties.

Compared to Figure 6a,b, it can be seen that there was a significant change in the copper sheet surface. It was obvious that the contaminant on the modified copper sheets was significantly reduced, indicating that the copper sheets modified with SA had the stronger self-cleaning ability.

To express quantitatively the anti-fouling properties of copper sheets surface of the different L_0_. The copper sheets ground with different grit sandpapers were immersed in the red suspension of the uniformly mixed kaolin-Rhodamine B.

**Figure 7:**
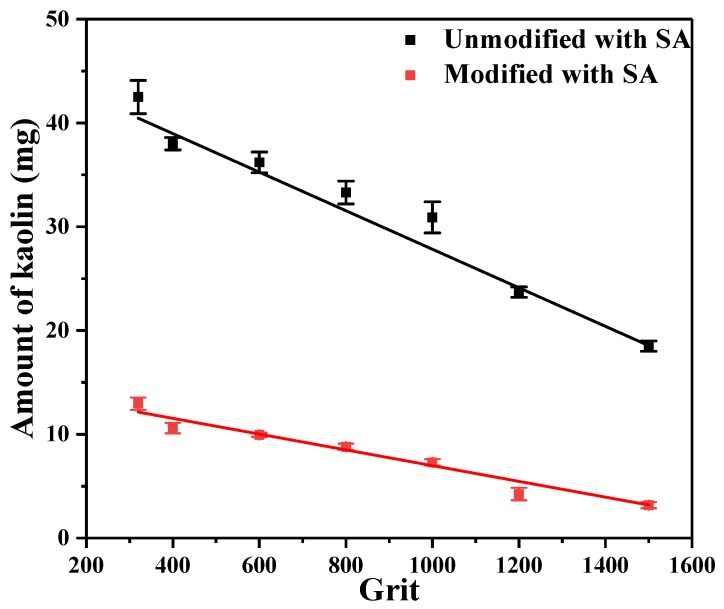
The self-cleaning performance of copper sheets ground with different grit SiC sandpapers.

The amount of kaolin adhered to the surface of unmodified and modified with SA copper sheets with different L_0_ was shown in Figure 7. It can be seen that as the sandpaper grit of ground copper sheets increased, the unit width L_0_ of the parabolic gradually decreased, and the amount of adsorbed kaolin on the copper sheets tended to decrease. In Figure 7, the adhered kaolin of the copper sheets modified with SA decreased. It was possible that as the surface energy of the copper sheets modified with SA decreased, the contact angle increased, the adhesion ability of the kaolin was lowered, and the stronger self-cleaning performance was.

In Figure 7, it can be seen that as the L_0_ on the copper sheets surface gradually decreased, the adhesion of kaolin to the copper sheets decreased. It can be seen that with a decrease of L_0_, the contact angle became higher and so was the self-cleaning. However, the surface adsorption capacity of the modified copper sheet was significantly less than that of the unmodified.

The unit width L_0_ of the parabolic on the copper sheet decreased gradually, the contact angle increased gradually, the amount of adsorption of the kaolin reduced gradually. The self-cleaning performance was gradually enhanced. In summary, there was a negative correlation between the reduction of L_0_ and self-cleaning performance.

## 4. Conclusions

In summary, by controlling grinding, chemical etching, oxidation, and modifying conditions, the parabolic structure can be obtained on the surface of the copper sheets and copper mesh. Copper mesh and copper sheets were used in oil–water separation, anti-fouling, and self-cleaning experiments, which showed that the parabolic morphology has great hydrophobic and anti-fouling properties.

The unit width L_0_ of the parabolic ground with 320, 800, and 1500 grit SiC sandpapers was 3.3 μm, 2.4 μm, and 1.6 μm, respectively, and the static contact angles modified with SA were 94.5°, 100.2°, and 110.5°. The oil–water separation efficiency of the copper meshes were 85%, 89%, and 94%, respectively.

Experimental results showed that the smaller the L_0_, the larger the contact angle, the better the hydrophobic performance, the higher the oil–water separation efficiency, and the better the self-cleaning performance. The unit width of the parabolic L_0_ was reduced and the hydrophobicity and the anti-fouling property were more easily realized.

The change of L_0_ can reflect the change in contact angle. It can be used as a significant indicator of hydrophobicity, self-cleaning, and oil–water separation efficiency in predicting the hydrophobicity and antifouling of materials.

## Figures and Tables

**Table 1:** Water contact angles of different copper sheets.

Different Situations	L_0_		
	3.2 μm	2.4 μm	1.6 μm
Unmodified with SA	68.7°	72.5°	82.4°
Modified with SA	94.5°	100.2°	110.5°

## References

[1] Chambers L.D., Stokes K.R., Walsh F.C., Wood R.J.K. (2006). Modern approaches to marine antifouling coatings. Surf. Coat. Technol..

[2] Sethi S.K., Manik G. (2018). Recent progress in super hydrophobic/hydrophilic self-cleaning surfaces for various industrial applications: A review. Polym.-Plast. Technol. Eng..

[3] Feng L., Li S.H., Li Y.S., Li H.J., Zhang L.J., Zhai J., Song Y.L., Liu B.Q., Jiang L., Zhu D.B. (2003). Super-hydrophobic surfaces: From natural to artificial. Adv. Mater..

[4] Woodward I., Schofield W.C.E., Roucoules V., Badyal J.P.S. (2003). Super-hydrophobic surfaces produced by plasma fluorination of polybutadiene films. Langmuir.

[5] Basheer S., Rashid N., Ashraf R., Akram M.S., Siddiqui M.A., Imanaka T., Akhtar M. (2017). Identification of a novel copper-activated and halide-tolerant laccase in Geobacillus thermopakistaniensis. Extremophiles.

[6] Jain R., Pitchumani R. (2018). Fabrication and characterization of zinc based superhydrophobic coatings. Surf. Coat. Technol..

[7] Genzer J., Efimenko K. (2006). Recent developments in superhydrophobic surfaces and their relevance to marine fouling: A review. Biofouling.

[8] Marmur A. (2006). Super-hydrophobicity fundamentals: Implications to biofouling prevention. Biofouling.

[9] Biehl P., von der Lühe M., Dutz S., Schacher F.H. (2018). Synthesis, characterization, and applications of magnetic nanoparticles featuring polyzwitterionic coatings. Polymers.

[10] Ilcíková M., Tkác J., Kasák P. (2015). Switchable Materials Containing Polyzwitterion Moieties. Polymers.

[11] Shirtcliffe N.J., Mchale G., Newton M.I., Zhang Y. (2009). Superhydrophobic Copper Tubes with Possible Flow Enhancement and Drag Reduction. ACS Appl. Mater. Interfaces.

[12] Shi F., Niu J., Liu J.L., Liu F., Wang Z.Q., Feng X.Q., Zhang X. (2010). Towards understanding why a superhydrophobic coating Is needed by water striders. Adv. Mater..

[13] Otten A., Herminghaus S. (2004). How plants keep dry: A physicist’s point of view. Langmuir.

[14] Quéré D. (2005). Non-sticking drops. Rep. Prog. Phys..

[15] Colangiuli D., Lettieri M., Masieri M., Calia A. (2018). Field study in an urban environment of simultaneous self-cleaning and hydrophobic nanosized TiO2-based coatings on stone for the protection of building surface. Sci. Total Environ..

[16] Dittrich P.S., Manz A. (2006). Lab-on-a-chip: Microfluidics in drug discovery. Nat. Rev. Drug Discov..

[17] Matosevic S., Szita N., Baganz F. (2011). Fundamentals and applications of immobilized microfluidic enzymatic reactors. J. Chem. Technol. Biotechnol..

[18] Nishino T., Meguro M., Nakamae K., Matsushita M., Ueda Y. (1999). The Lowest Surface Free Energy Based on −CF3 Alignment. Langmuir.

[19] Young T. (1800). An Essay on the Cohesion of Fluids. Philos. Trans. R. Soc. Lond..

[20] Wenzel R.N. (1936). Resistance of solid surfaces to wetting by water. Ind. Eng. Chem..

[21] Cassie A.B.D., Baxter S. (1944). Wettability of porous surfaces. Trans. Faraday Soc..

[22] Shi Z., Liu Z., Song H., Zhang X. (2016). Prediction of contact angle for hydrophobic surface fabricated with micro-machining based on minimum Gibbs free energy. Appl. Surf. Sci..

[23] Salvadori M.C., Cattani M., Oliveira M.R.S., Teixeira F.S., Brown I.G. (2010). Design and fabrication of superhydrophobic surfaces formed of microcavities. Appl. Phys. Lett..

[24] Bittoun E., Marmur A. (2009). Optimizing Super-Hydrophobic Surfaces: Criteria for Comparison of Surface Topographies. J. Adhes. Sci. Technol..

[25] Nosonovsky M. (2007). Model for solid-liquid and solid-solid friction of rough surfaces with adhesion hysteresis. Chem. Phys..

[26] Yamamoto K., Ogata S. (2008). 3-D thermodynamic analysis of superhydrophobic surfaces. J. Colloid Interface Sci..

[27] Qian Z. (2017). Prediction for Super-Hydrophobic Performance and Oil/Water Separation Applications for the Material of Mesh Membrane. Master’s Thesis.

[28] Gao X., Yan X., Yao X., Xu L., Zhang K., Zhang J., Yang B., Jiang L. (2007). The Dry-Style Antifogging Properties of Mosquito Compound Eyes and Artificial Analogues Prepared by Soft Lithography. Adv. Mater..

[29] Zhang M., Wu Z., Meng F., Lin H. (2019). Facile preparation of grass-like hierarchical structured Γ-AlOOH coated stainless steel mesh with superhydrophobic and superoleophilic for highly efficient oil-water separation. Sep. Purif. Technol..

[30] Long Y., Shen Y., Tian H., Yang Y., Feng H., Li J. (2018). Superwettable Coprinus comatus coated membranes used toward the controllable separation of emulsified oil/water mixtures. J. Membr. Sci..

[31] Kang H., Cheng Z., Lai H., Ma H., Liu Y., Mai X., Wang Y., Shao Q., Xiang L., Guo X. (2018). Superlyophobic anti-corrosive and self-cleaning titania robust mesh membrane with enhanced oil/water separation. Sep. Purif. Technol..

[32] Ren G., Song Y., Li X., Zhou Y., Zhang Z., Zhu X. (2018). A superhydrophobic copper mesh as an advanced platform for oil-water separation. Appl. Surf. Sci..

[33] Chen C., Du C., Weng D., Mahmood A., Feng D., Wang J. (2018). Robust Superhydrophobic polytetrafluoroethylene nanofibrous coating fabricated by self-assembly and its application for oil/water separation. ACS Appl. Nano Mater..

[34] Jiang B., Zhang H., Zhang L., Sun Y., Xu L., Sun Z., Gu W., Chen Z., Yang H. (2017). Novel one-step, in situ thermal polymerization fabrication of robust superhydrophobic mesh for efficient oil/water separation. Ind. Eng. Chem. Res..

[35] Li J., Xu C., Zhang Y., Wang R., Zha F., She H. (2016). Robust superhydrophobic attapulgite coated polyurethane sponge for efficient immiscible oil/water mixture and emulsion separation. J. Mater. Chem. A.

[36] Xue C.H., Guo X.J., Zhang M.M., Ma J.Z., Jia S.T. (2015). Fabrication of robust superhydrophobic surfaces by modification of chemically roughened fibers via thiol-ene click chemistry. J. Mater. Chem. A.

[37] Li C., Wu L., Yu C., Dong Z., Jiang L. (2017). Peristome-Mimetic curved surface for spontaneous and directional separation of micro water-in-oil drops. Angew. Chem..

[38] Liu G., Wang J., Wang W., Yu D. (2019). A novel PET fabric with durable anti-fouling performance for reusable and efficient oil-water separation. Colloids Surf. A.

[39] Li N., Wu L., Yu C., Dai H., Wang T., Dong Z., Jiang L. (2018). Ballistic jumping drops on superhydrophobic surfaces via electrostatic manipulation. Adv. Mater..

[40] Wang T., Si Y., Luo S., Dong Z., Jiang L. (2019). Wettability manipulation of overflow behavior via vesicle surfactant for water-proof surface cleaning. Mater. Horiz..

[41] Wang Q., Dai B., Bai J., Yang Z., Guo S., Ding Y., Yang L., Lei P., Han J., Zhu J. (2016). Synthesis of vertically aligned composite microcone membrane filter for water/oil separation. Mater. Des..

[42] Zang D., Liu F., Zhang M., Niu X., Gao Z., Wang C. (2015). Superhydrophobic coating on fiberglass cloth for selective removal of oil from water. Chem. Eng. J..

[43] Qiao X., Yang C., Zhang Q., Yang S., Chen Y., Zhang D., Yuan X., Wang W., Zhao Y. (2018). Preparation of parabolic superhydrophobic material for oil-water separation. Materials.

[44] Wu D., Wang P., Wu P., Yang Q., Liu F., Han Y., Xu F., Wang L. (2015). Determination of contact angle of droplet on convex and concave spherical surfaces. Chem. Phys..

[45] Zhao J., Su Z., Yan S. (2015). Thermodynamic analysis on an anisotropically superhydrophobic surface with a hierarchical structure. Appl. Surf. Sci..

[46] Luo B.H., Shum P.W., Zhou Z.F., Li K.Y. (2010). Surface geometrical model modification and contact angle prediction for the laser patterned steel surface. Surf. Coat. Technol..

[47] Nickelsen S., Moghadam A.D., Ferguson J.B., Rohatgi P. (2015). Modeling and experimental study of oil/water contact angle on biomimetic micro-parallel-patterned self-cleaning surfaces of selected alloys used in water industry. Appl. Surf. Sci..

[48] Cao Z.F., Wang J., Qiu P., Yang F., Wang S., Liu G., Zhong H. (2018). Hydrophobic coatings for improving corrosion resistance of manganese substrate. Surf. Coat. Technol..

[49] Liu G.H., Zhou B.H., Li Y.F., Qi T.G., Li X.B. (2015). Surface properties of superfine alumina trihydrate after surface modification with stearic acid. Int. J. Miner. Metall. Mater..

[50] Ma Q., Cheng H., Fane A.G., Wang R., Zhang H. (2016). Recent Development of advanced materials with special wettability for selective oil/water separation. Small.

[51] Liu Y.Q., Zhang Y.L., Fu X.Y., Sun H.B. (2015). Bioinspired underwater superoleophobic membrane based on a graphene oxide coated wire mesh for efficient oil/water separation. ACS Appl. Mater. Interfaces.

[52] Lin X., Choi M., Heo J., Jeong H., Park S., Hong J. (2017). Cobweb-Inspired superhydrophobic multiscaled gating membrane with embedded network structure for robust water-in-oil emulsion separation. ACS Sustain. Chem. Eng..

[53] Cao C., Jiang C. (2018). Fabrication of robust surfaces with special wettability on porous copper substrates for various oil/water separations. Chem. Eng. J..

[54] Zhang D., Li L., Wu Y., Sun W., Wang J., Sun H. (2018). One-step method for fabrication of superhydrophobic and superoleophilic surface for water-oil separation. Colloids Surf. A Physicochem. Eng. Asp..

